# Designing Digital Mental Health Support for Paramedics Exposed to Trauma: Qualitative Study of Lived Experiences and Design Preferences

**DOI:** 10.2196/76158

**Published:** 2025-09-12

**Authors:** Nicola Cogan, Spence Whittaker, Ashleigh Craig, Lucy Milligan, Robyn McCluskey, Tara Burns, Alison Kirk, Susan Rasmussen, William Hodgson

**Affiliations:** 1 Department of Psychological Sciences and Health, University of Strathclyde, Graham Hills Building, Glasgow, G1 1XQ, United Kingdom

**Keywords:** paramedics, trauma, digital mental health, first responders, qualitative research, usability, peer support, occupational stress

## Abstract

**Background:**

Paramedics face frequent exposure to trauma and intense occupational stress, often under conditions of limited psychological support and ongoing stigma. Digital mental health interventions have the potential to offer accessible, confidential, and tailored support. However, their acceptability and design must be informed by the lived experiences of paramedics to ensure effectiveness.

**Objective:**

This study aimed to explore the experiences of trauma exposure among UK paramedics in the workplace and their views on the design and delivery of digital mental health interventions.

**Methods:**

Semi-structured interviews were conducted with 22 UK paramedics. Participants were recruited through purposive and snowball sampling. Interviews were transcribed verbatim and analyzed using reflexive thematic analysis. Ethical approval was obtained, and trauma-informed principles were applied throughout data collection and analysis.

**Results:**

Five key themes were identified: (1) It Has to Feel Easy to Use*:* highlighting the need for digital tools that reduce cognitive burden and are accessible during unpredictable shifts; (2) Make It Fit My Needs: calling for interventions specifically designed for paramedics, with lived-experience-informed language and delivery; (3) We Need to Talk to Each Other: describing a strong desire for peer connection while recognizing barriers such as stigma and shift pressures; (4) I Need to Know It’s Safe: emphasizes the importance of anonymity, data privacy, and psychological safety; and (5) Support Needs to Feel Human: reinforcing the value of integrating digital tools with human connection and professional services. Participants expressed strong support for an app-based solution that offers anonymity, rapid accessibility, and flexibility, while preserving opportunities for human interaction.

**Conclusions:**

Paramedics face unique mental health challenges that are not adequately addressed by existing services. Digital mental health tools offer promise if they are carefully co-designed to reflect the realities of frontline work. Anonymity, usability, peer connection, and integration with existing support systems are critical to engagement. These findings offer actionable insights for the development of trauma-informed, context-sensitive digital mental health interventions for emergency service workers.

## Introduction

Frontline workers, including paramedics, firefighters, police officers, and emergency health care staff, operate in some of the most high-pressure environments, frequently encountering life-threatening emergencies, traumatic incidents, and high-stress situations [1,2]. The cumulative exposure to critical incidents places these workers at significant risk of developing mental health conditions such as posttraumatic stress disorder, anxiety, depression, and burnout [3,4]. While all frontline workers experience occupational stress, research consistently shows that paramedics report some of the poorest mental health outcomes among emergency responders, including elevated levels of psychological distress, emotional exhaustion, and suicidal ideation compared to their counterparts in firefighting and law enforcement [5,6].

Paramedics face unique challenges that exacerbate their vulnerability to mental health difficulties [7]. They frequently operate under relentless workloads with little opportunity for recovery between challenging and often traumatic calls. Paramedic work requires responding to unpredictable situations with limited resources at any hour of the day. In these high-pressure, uncontrolled settings, paramedics are often required to make complex decisions while also managing the effects of sleep deprivation and disrupted circadian rhythms caused by shift work [8,9]. The unpredictability of their role, such as never knowing the severity of an incident before arriving on the scene, further compounds stress levels. Additionally, paramedics often work in isolated environments, providing care in ambulances, homes, or public settings without immediate access to team support [10]. This lack of structured debriefing opportunities can lead to cumulative emotional distress, as paramedics must frequently suppress their emotions to remain professional in high-stakes situations [11].

Another significant challenge is moral injury, a psychological distress experienced when actions taken in the line of duty conflict with personal values or ethical standards [12]. Paramedics frequently witness unavoidable suffering, resource limitations, and cases where they are unable to save a life, leading to profound feelings of guilt, self-doubt, and helplessness [13]. Furthermore, paramedics often provide end-of-life care in uncontrolled environments, making their experiences emotionally intense and personally distressing [14]. This repeated exposure to human suffering, combined with high-performance expectations and limited emotional support, places them at heightened risk of long-term psychological harm [15].

In addition to physical trauma cases, paramedics are frequently dispatched to mental health-related emergencies, including suicide attempts and death by suicide, self-harm incidents, and acute psychiatric crises [16]. These callouts present unique stressors, as paramedics must navigate complex interactions with individuals in extreme distress, often with limited mental health training or specialist support [17,18]. Attending suicide-related incidents has been shown to significantly impact paramedics’ mental well-being, particularly when required to interact with grieving families or manage high-risk patients without adequate resources [19]. The emotional toll of these callouts, combined with the stigma surrounding mental health within emergency services, contributes to burnout, compassion fatigue, and secondary traumatic stress among paramedics [20].

Another critical issue paramedics face is exposure to workplace violence and assaults. Paramedics frequently encounter aggressive patients, intoxicated individuals, or bystanders who may become physically or verbally abusive [21]. This occupational hazard is further exacerbated by working alone or in unpredictable environments where immediate backup may not be available. Exposure to workplace violence not only increases the risk of physical injury but also significantly contributes to long-term psychological distress, fear, and hypervigilance [22]. Studies have shown that repeated exposure to aggression and violence leads to elevated rates of posttraumatic stress disorder, anxiety, and depressive symptoms among paramedics, further compounding the already high levels of occupational stress they experience [10,23,24]. Despite policies aimed at protecting emergency responders, many paramedics feel unsupported when reporting incidents of violence, contributing to a culture of normalization and underreporting [25].

Despite these challenges, many paramedics do not seek professional mental health support due to stigma, fear of judgment, and concerns about confidentiality [26]. Prior research has identified that paramedics are less likely than other emergency responders to disclose psychological distress, fearing that such admissions could lead to professional consequences, social isolation, or doubts about their fitness to work [11]. Traditional face-to-face mental health support services, while available, remain underused due to perceived stigma, logistical barriers, and a culture that often prioritizes resilience over emotional well-being [27].

Such issues highlight the urgent need for tailored mental health support for paramedics [28]. Digital mental health interventions present a promising avenue for overcoming barriers to traditional face-to-face support by offering accessible, confidential, and flexible options for managing psychological distress [29]. However, for such interventions to be effective, they must be designed with direct input from end users to ensure relevance and usability [30]. Cocreation is increasingly recognized as a critical component in the development of digital mental health interventions, ensuring they are relevant, engaging, and responsive to the needs of the end user [31]. By involving paramedics in the design process, digital interventions can be tailored to align with their unique work environments, stressors, and coping mechanisms. Research has shown that interventions developed in collaboration with users have higher adoption rates and greater long-term efficacy compared to those designed without user input [32]. For paramedics, cocreation is particularly vital as they face distinct challenges such as exposure to trauma, high-pressure decision-making, and limited opportunities for structured mental health support. Generic digital interventions may fail to address the realities of their work, leading to disengagement and limited effectiveness. Additionally, cocreation fosters a sense of ownership and trust in the intervention, reducing potential stigma associated with help-seeking. When end users see their insights reflected in the final digital intervention, they are more likely to perceive the intervention as relevant and beneficial to their well-being [33]. Given the demanding and often unpredictable nature of paramedic work, it is crucial that digital mental health interventions are not only evidence-based but also practical and person-centered.

This study aims to explore paramedics’ perspectives on digital mental health interventions and identify key design considerations to inform the development of tailored support systems [34]. It seeks to understand paramedics’ experiences with workplace trauma and their perspectives on the acceptability of a digital mental health intervention. The research also aims to contribute to the growing body of literature on digital mental health interventions for frontline workers. This study addresses the following research questions:

1. What are paramedics’ experiences with workplace trauma, and how do they currently manage their mental health?
2. What features and functionalities do paramedics consider essential for a digital mental health intervention?

## Methods

### Study Design

This study used a qualitative design using in-depth, semi-structured interviews to explore paramedics’ experiences with workplace trauma and their perspectives on digital mental health interventions. A qualitative approach was chosen to generate detailed insights into participants’ mental health needs and intervention preferences, allowing for a deeper understanding of lived experiences that quantitative methods may not capture. Grounded in a constructivist epistemology, this approach emphasizes the coconstruction of meaning between researchers and participants and acknowledges the active role of the researcher in interpreting the data. It aligns with the participatory and trauma-informed principles underpinning this study, ensuring sensitivity to the emotional content of interviews and valuing the voices of participants throughout the analytic process [35].

### Recruitment

Recruitment was conducted through professional networks, paramedic organizations, and web-based forums to ensure a diverse sample that captured a broad range of experiences within emergency medical services. A combination of purposive and snowball sampling was used. Purposive sampling enabled the targeted recruitment of participants with relevant professional backgrounds and trauma exposure, while snowball sampling allowed existing participants to refer colleagues with similar experiences, broadening the reach and diversity of the sample. Recruitment strategies included direct invitations disseminated via paramedic organizations, participant referrals through word-of-mouth, and digital flyers shared on professional social media platforms. Individuals were eligible to participate if they resided in the United Kingdom, were aged 18 years or older, had been employed in a paramedic role for at least 6 months, and self-identified as having experienced trauma in the workplace at the time of this study. Interested participants were provided with an electronic participant information sheet detailing this study’s purpose, procedures, and ethical safeguards. Following written informed consent, interviews were scheduled at times convenient to each participant.

All interviews were conducted via Microsoft Teams to ensure accessibility, data security, and consistency in data collection [36]. A total of 22 paramedics working in emergency medical services were recruited through purposive and snowball sampling [37]. This sample size was sufficient as qualitative research prioritizes depth, richness, and variation in experiences over numerical representativeness [38]. Studies on first responders and trauma have drawn meaningful conclusions from similar sample sizes [39-41], demonstrating that a focused cohort can yield transferable, contextually grounded insights [42]. Purposive sampling ensured that participants had direct lived experience relevant to the research aims, while snowball sampling enhanced inclusivity by recruiting those hesitant to engage in trauma-related research [43,44].

### Data Collection

A semi-structured interview guide was developed to facilitate in-depth exploration of participants’ experiences, drawing on previous research related to occupational trauma and mental health in emergency services [45,46]. The guide was designed to prompt discussion on key areas, including encounters with workplace trauma, access to and experiences with existing mental health support, perceived organizational and cultural barriers to seeking help, and preferences for digital mental health interventions tailored to paramedic needs. Questions were open-ended and designed to elicit rich, narrative data while allowing for flexibility based on individual experiences. The guide was refined iteratively during the early phases of data collection, with minor modifications made to reflect emerging themes and to enhance sensitivity to the language and challenges specific to the paramedic context. This adaptive approach ensured the interviews remained responsive to participants’ perspectives while maintaining consistency across core thematic areas. All interviews were conducted remotely using Microsoft Teams, allowing for flexible scheduling and increased accessibility for participants working variable shift patterns. This web-based format also aligned with trauma-informed practices by enabling participants to choose a safe and private environment in which to share their experiences.

Interviews were conducted over 18 months and ranged in length from approximately 40 to 75 minutes (mean 58.2, SD 10.2). All interviews were conducted remotely, allowing participants to join from a private setting of their choice. The final sample size (n=22) was determined based on the richness, relevance, and diversity of the data, consistent with the principles of reflexive thematic analysis [42]. Rather than aiming for data saturation, we drew on the concept of information power to guide sample sufficiency, prioritizing depth, variation, and the capacity of the data to meaningfully address the research questions. The dataset was judged to provide rich, nuanced insights into paramedics’ lived experiences of workplace trauma and preferences for digital support, with recurring patterns and interpretative depth evident across the interviews.

### Ethical Considerations

This study forms part of a broader program of research exploring trauma exposure and psychological support needs among frontline workers in the United Kingdom. It specifically focuses on the views and experiences of paramedics regarding the acceptability and design of digital mental health interventions, with distinct aims and analyses from prior related research [28]. Given the sensitive nature of workplace trauma, ensuring the well-being and safeguarding of participants was a key ethical consideration. This study received ethical approval from the University of Strathclyde Ethics Committee (UEC22/92). Informed consent was obtained before participation, and ethical approval was granted by the institutional review board. Participants were informed of their right to withdraw at any stage without repercussions. Data were anonymized and stored securely, per GDPR and institutional data protection policies, to protect participant identity and confidentiality. Participants were offered a £20 (US $23.29) gift voucher as a thank-you for their time and contribution to the interviews. Additionally, signposting to mental health support services was provided to all participants, ensuring access to professional help if discussing traumatic experiences triggered distress [47]. Researching trauma-related topics requires careful ethical handling, and efforts were made to create a psychologically safe space where participants could share their experiences without fear of judgment or harm [48]. This study also emphasized the importance of participatory methods and lived experience perspectives in shaping digital mental health interventions. By centering paramedics’ voices, this study aligns with the principles of participatory research, ensuring that digital interventions are not only theoretically sound but also practically applicable to frontline workers’ needs. Additionally, researcher well-being was considered, given the emotionally demanding nature of trauma-related research. The research team received specific training, led by a Health and Care Professions Council–registered clinical psychologist, on conducting sensitive research and applying trauma-informed principles. This included prioritizing self-care, reflexive journaling throughout the research process, and recognizing signs of secondary trauma exposure. To further safeguard well-being, interviewers were limited to 1 interview per day, followed by a structured debrief with the research lead. Additionally, a structured debriefing process was implemented to provide access to professional supervision and peer support [49,50]. Reflexive meetings were held regularly throughout the research process, fostering a team approach to addressing challenges, ensuring ethical consistency, and supporting both participant and researcher well-being [51,52].

### Analysis

All interviews were one-to-one and were audio-recorded and transcribed in full verbatim. The data were then managed in NVivo (version 12, QSR International) and analyzed using a reflexive thematic approach, following the framework by Braun and Clarke [53], which is particularly suited for studies exploring complex psychological and social phenomena [54]. This approach builds upon the earlier thematic analysis framework of Braun and Clarke [55], adopting a more explicitly reflexive and interpretative approach. It emphasizes the researcher’s active role in meaning-making [56]. Themes are not viewed as pre-existing within the data but are constructed through deep engagement and iterative interpretation [57]. This began with the process of familiarization and immersion within the data, generating initial codes organically and fluidly. Initial codes were logged, with records of how each theme evolved through discussion and refinement. Regular team meetings were held to review and challenge coding decisions, ensuring that the analysis was grounded in the data while allowing for flexibility in interpretation. Any discrepancies in theme development were resolved through collaborative discussion in the research team, ensuring that final themes were coconstructed rigorously and reflexively [58].

A value-based approach to qualitative research was applied, ensuring a commitment to ethical transparency, reflexivity, and participant-centered analysis [59]. This framework emphasizes the importance of researcher subjectivity and the co-construction of knowledge, aligning with the study’s focus on participatory research and lived experience perspectives. Recognizing the limitations of rigid, universal evaluative tools for qualitative research, the Big Q Qualitative Reporting Guidelines were considered to ensure that methodological choices aligned with qualitative research values rather than externally imposed standards rooted in postpositivist traditions [59]. This framework prioritizes methodological congruence, reflexivity, and the interactive nature of knowledge production. To ensure transparency and rigor in the research process, an audit trail was maintained throughout data collection, analysis, and interpretation. This involved systematically documenting all key decisions made at each stage of this study, providing a structured record of the rationale behind methodological choices and analytic interpretations.

## Results

### Participant Characteristics

The final sample consisted of 22 paramedics. Participants ranged in age from 23 to 59 years (mean 38.22, SD 9.28), with professional experience ranging from 1 to 38 years (mean 13.11, SD 6.93). Thirteen (59%) participants identified as male and 9 (41%) as female. The majority (82%, n=18) identified as White British, while 4 (18%) participants identified as belonging to ethnic minority backgrounds. Most participants were based in England (64%, n=14), with the remainder in Scotland (36%, n=8). All participants self-identified as having experienced occupational trauma that had negatively impacted their mental health and well-being. See Table 1 for a full breakdown of demographic characteristics.

**Table 1.** Participant characteristics. Names are pseudonyms. Ethnicity categories adapted from UK Office for National Statistics classifications.

Name	Gender	Age (years)	Service (years)	Ethnicity
Billy	Male	30	5	White British
Harry	Male	34	10	White Scottish
Gillian	Female	47	21	White British
Garry	Male	51	10	White Scottish
Mike	Male	40	2	White Scottish
Jan	Male	28	3	White Other (Polish)
Nathan	Male	50	21	White Irish
Terry	Female	44	17	White Scottish
Sam	Male	51	15	White British
William	Male	39	5	White Scottish
James	Male	37	12	White British
Rachel	Female	36	11	White Scottish
Paul	Male	45	20	White Irish
Laura	Female	33	8	White Scottish
Jane	Female	48	25	White British
Emma	Female	29	6	White British
David	Male	41	14	White Scottish
Sarah	Female	42	19	White British
Leigh	Female	46	23	White Scottish
Eric	Male	50	18	White Other (Polish)
Rebecca	Female	35	9	White British
Luke	Male	38	16	White British

### Themes

The themes presented below reflect the diverse and often deeply personal ways in which paramedics make sense of their experiences of occupational trauma. Given the subjective and multifaceted nature of psychological trauma, participants were invited to self-define their experiences, enabling the analysis to capture a broad spectrum of emotional and psychological responses. This approach respected individual interpretations of what constitutes trauma and ensured that the analysis remained grounded in participants’ own meanings and language. Five key themes emerged from the analysis, capturing the experiences and preferences of paramedics regarding digital mental health interventions. These themes illustrate their needs for usability, personalization, social support, privacy, and human connection (Table 2).

**Table 2.** Summary of themes identified in the thematic analysis.

Theme	Description
It Has to Feel Easy to Use—Navigating Acceptability and Usability	Intervention must be simple and intuitive. Should reduce cognitive load postshift. Quick access is crucial for usability.
Make It Fit My Needs—The Importance of Customization and Personalization	Tools should be tailored to paramedic experiences. Generic interventions were seen as ineffective. Relevance drives engagement.
We Need to Talk to Each Other—Social Connection and Peer Support	Peer support is highly valued. Digital tools can enable safe, structured connection. Reduces isolation and builds validation.
I Need to Know It’s Safe—Confidentiality, Privacy, and Safety	Concerns about stigma and data breaches. Security and trust are prerequisites for use. Anonymity must be guaranteed.
Support Needs to Feel Human—Integration With Existing Services	Digital tools should complement, not replace, human support. Facilitate access to professional help. Include guided check-ins and support pathways.

### Theme 1: It Has to Feel Easy to Use—Navigating Acceptability and Usability

Acceptability was closely linked to usability for many participants, who emphasized that any digital intervention must feel simple, nonintrusive, and easy to engage with during or after demanding shifts. Paramedics described the cognitive burden of their work, noting that complex or time-consuming tools would be impractical in high-pressure environments. Many highlighted that their workload and unpredictable schedules often leave little energy for engaging with structured mental health support.

*Getting five minutes to breathe is rare now. We used to have time to gather ourselves, but not anymore.*

[Rachel]

*You deal with something traumatic, but before you’ve processed it, you’re already at the next job.*

[Luke]

These accounts reflect the high workload pressures paramedics face, with limited opportunities for decompression or reflection. Digital interventions, therefore, were seen as acceptable only if they were intuitive, low-effort, and seamlessly integrated into the rhythm of shift-based work.

*You can’t go through life just dealing with trauma on adrenaline… I have to have a way of bringing that down and managing that.*

[Paul]

Some paramedics reported becoming emotionally desensitized over time, reflecting a gradual numbing that made it harder to recognize when support was needed.

*It’s trauma after trauma. Eventually it just becomes normal, and that’s when you realise you’ve stopped feeling it.*

[Leigh]

This emotional distancing may contribute to reduced help-seeking unless support is easily accessible and presented in a way that feels informal and manageable.

Participants expressed that traditional face-to-face mental health support often felt inaccessible, both logistically and psychologically, due to stigma and confidentiality concerns. In contrast, digital interventions were viewed as potentially acceptable because of their on-demand nature and discretion.

*I wouldn’t go to a therapist, even if I needed to. It’s just not what we do. But if I could check in with something that doesn’t feel like a big deal, I’d probably use it.*

[Nathan]

*Going through work to get help feels risky. But if I can do it quietly, that changes everything.*

[Rebecca]

A key factor influencing acceptability was that the intervention should not require additional cognitive or emotional effort, particularly at the end of long or emotionally draining shifts.

*If I’ve had a long shift, I don’t want something that’s going to take brain power. It has to be quick and easy.*

[Jane]

Some participants also saw digital tools as a proactive way to manage stress before it became overwhelming, valuing short, in-the-moment strategies.

*Even a short breathing exercise or prompt could stop it spiralling after a tough job.*

[Laura]

In sum, usability was seen as foundational to acceptability. Tools that were easy to navigate, unobtrusive, and offered brief, practical support were more likely to be welcomed and used.

### Theme 2: Make It Fit My Needs—the Importance of Customization and Personalization

Beyond usability, participants consistently emphasized the importance of personal relevance and individual fit in shaping how acceptable an intervention would feel. In this context, acceptability was not just about simplicity or ease of use, but about feeling seen, understood, and supported in a way that resonated with paramedics’ specific experiences. When discussing the acceptance and needs for an appropriate intervention, participants noted the importance of “specific” [Rachel] help, comprising needs unique to paramedics. It was noted that others “just don’t get it” [Leigh].

Participants felt that credible support should be tailored to paramedics specifically, as they felt they are “different from other first responders” [Eric] and “other members of society” [Rebecca]. Participants stated that creators of an intervention should have a strong knowledge and preferably, experience of life as a paramedic, which can be evidenced by comments such as:

*You need people designing this who understand our reality. Otherwise, it just won’t connect.*

[Nathan]

*There’s no substitute for lived experience. We spot it straight away when something feels off.*

[Emma]

Participants viewed themselves as different from other occupations, given the nature of their working environment, and therefore stated they required an intervention “that’s tailored for an individual” [Leigh]. All participants stated that they would be more likely to speak of mental health concerns and seek help if they could speak to someone with “real insight into the job” [Rachel].

Additionally, participants suggested they were more likely to access help if it could be “completely anonymous” [Eric], given the stigmatization prevalent within the occupation:

*If people don’t know it’s you, you’re far more likely to be honest.*

[David]

Regarding anonymity, all participants suggested that an identifiable method of intervention would prevent them from accessing help. Overall, acceptability was high across participants for an intervention, with all participants stating they would be accepting of an anonymous and specific intervention:

*Even something small, like a quick check-in after a shift, if it’s anonymous and for us, I’d use it.*

[Laura]

*If nothing changes, we’re just going to lose more people. This kind of support has to be built in.*

[Rachel]

Participants reflected the timely need for an intervention comprising the detailed characteristics to prevent “experienced people from burning out and leaving for good” [Emma]. Hence, all participants suggested that a digital intervention in app form would be beneficial, given that it could be specific to them, constantly accessible, and confidential, as voiced by Eric:

*Having it on our phones would make it feel like a normal part of the job (pauses) something we all use, not something secret or awkward.*

[Eric]

Furthermore, in discussing potential technological barriers, participants described digital tools as familiar and well-integrated into their working lives, with 1 noting it was “second nature now” [Sam]. As such, acceptability was not about the presence of technology per se, but about how well it aligned with paramedics’ specific needs and values:

*We use tech every day. It’s not strange anymore. If anything, it makes sense to use it for our mental health too.*

[Rebecca]

Participants emphasized that interventions were more likely to be accepted if they could be tailored based on personal needs, emotional state, or shift patterns. This capacity for customization fostered a sense of personal relevance and ownership, making the intervention feel trustworthy and worth engaging with:

*You’re more likely to keep going back if it feels like it’s built for you.*

[Sarah]

### Theme 3: We Need to Talk to Each Other—Social Connection and Peer Support

A strong theme that emerged was the need for social connection, particularly among colleagues who understand the unique pressures of the role. Participants spoke about how workplace trauma is often managed informally through discussions with peers, yet increasing work demands have reduced these opportunities. For instance, 1 paramedic shared how important it was to debrief but noted that opportunities to do so were limited:

*There are fewer and fewer chances to talk things through. That’s when you start carrying things on your own.*

[Luke]

However, some expressed concerns about the diminishing support culture in their work environment:

*The support bit, it’s just not there.*

[Leigh]

The need for peer support was reinforced by the recognition that sharing experiences with colleagues who “really get it” [Mike] provides comfort and validation:

*If you're involved, first responders and stuff, you have an understanding of what you’ve been through*

[David]

Despite this, some participants highlighted a reluctance to open up about their struggles, especially within a “macho culture” that discouraged vulnerability:

*You certainly wouldn’t admit it to your colleagues. It was a stiff upper lip culture, you know, just get on with it*

[Terry]

Given these concerns, digital interventions were seen as a potential way to facilitate peer connection in a safe and anonymous environment. Some participants suggested that a digital platform could provide structured opportunities to connect with others who have shared similar experiences, while still allowing individuals to maintain privacy:

*I wouldn’t want to sit in a room and talk about it, but if there was something online where you could connect with people who’ve been through the same, that would be different.*

[Rebecca]

Others highlighted the potential for digital interventions to offer moderated peer forums or guided group discussions, ensuring that support remains professional and constructive:

*It would have to be somewhere safe, not just a big free-for-all chat where people bring each other down. Maybe something that’s monitored, or with prompts to help guide discussions.*

[James]

The ability to share experiences without fear of judgment was seen as a crucial element in increasing engagement with peer support mechanisms. Digital tools that allow for anonymous peer check-ins or shared reflections were regarded as particularly valuable:

*Sometimes you don’t need someone to fix it, you just need to know someone else has been through it too. If an app could help with that, I’d use it.*

[Luke]

### Theme 4: I Need to Know It’s Safe—Confidentiality, Privacy, and Safety

A major concern for participants was data security and confidentiality. Many reported that stigma and fears of professional consequences prevented them from seeking help. The pressure to appear resilient and the fear of being judged or deemed unfit for their role created barriers to accessing mental health support. Participants highlighted that any digital intervention must prioritize absolute anonymity to encourage engagement.

*There’s a fear. There’s a big fear… About our professional registration, because you’ve got a mental health condition… because if you speak, you’re fired.*

[Gillian]

*There are people with the attitude that if you suffer from mental health problems, then this is obviously not a job for you… you’re clearly not cut out for it.*

[Garry]

Ensuring anonymity was seen as crucial, as paramedics were concerned that seeking support through formal channels might lead to professional repercussions or workplace stigma:

*You want to be sure it’s completely anonymous. That’s the only way I’d open up.*

[Jan]

Digital interventions were perceived as a potential solution, offering a confidential and secure platform where paramedics could access support without fear of being identified. Participants suggested that digital tools should include features such as encrypted communication, anonymous logins, and discreet access options to protect their privacy. Additionally, some paramedics expressed the need for clear information on how their data would be stored and who would have access to it:

*You’d have to be upfront about what happens with any data. If I’m putting anything in, I want to know it’s not going anywhere else.*

[Laura]

Several participants described how the long-term effects of trauma can be overwhelming, making it even harder to seek help. They acknowledged that while digital interventions could not replace professional therapy, they could provide an important first step for those who are hesitant to engage with traditional mental health services. By incorporating stringent privacy protections and offering an anonymous space for processing emotions, digital interventions were viewed as potentially bridging the gap for paramedics who might otherwise avoid seeking mental health support due to concerns about stigma and confidentiality.

### Theme 5: Support Needs to Feel Human—Integration With Existing Services

Although participants expressed a willingness to engage with digital interventions, they stressed that technology should not replace human-led mental health support. Many emphasized that digital tools should be used as a supplement rather than a substitute for direct peer or professional support. While digital interventions offer accessibility and confidentiality, participants acknowledged the value of human connection in processing trauma and stress. One paramedic highlighted how the lack of structured support left them feeling exposed:

*Sometimes it’s just a few words from a person that makes all the difference. The app can’t do it all.*

[Sarah]

Participants expressed a strong need for interventions that offer structured and ongoing support, ensuring that paramedics feel genuinely seen and supported in their mental health journey. They recognized that digital tools could help fill gaps in existing support services, but were cautious that such tools must integrate seamlessly with professional and peer-based systems.

*I think having a digital app for trauma is the right thing. I think building such an app, it’s something that needs to be built for the people that you’re wanting to use it.*

[Harry]

Many paramedics emphasized that for a digital intervention to be truly beneficial, it should facilitate bridging the gap between self-help and professional support. They suggested that digital tools could provide structured pathways to connect users with trained professionals when needed, ensuring that those who require more intensive intervention are not left without options:

*I’d use something like a digital app if I knew there was a way to reach out to a real person if I needed it. Just knowing that’s there makes a difference.*

[James]

Another key aspect raised by participants was the need for human elements within the digital space to create a sense of connection and validation. Some suggested that interventions could incorporate recorded messages from experienced paramedics or psychologists to guide them through difficult moments:

*If I could hear from someone who’s been in my shoes, even if it’s just pre-recorded guidance, that would feel more real than just reading generic advice.*

[David]

Participants also highlighted the need for structured check-ins and follow-ups to ensure ongoing support, rather than a 1-time or reactive approach:

*It shouldn’t just be something you use once when you’re in crisis. It should check in with you, like, “How are you doing?” and offer reminders to engage with it.*

[Emma]

These insights reinforce the idea that while digital interventions can provide essential support and accessibility, they should not function in isolation. Instead, they should act as an entry point into broader mental health care, offering both immediate coping strategies and seamless integration with existing services. By incorporating human-centered elements such as guided support, personalized check-ins, and links to professional care, digital tools can become a valuable and sustainable resource for paramedics navigating workplace trauma.

## Discussion

### Principal Findings

This study explored paramedics’ experiences of workplace trauma, their approaches to managing mental health, and their perspectives on the acceptability and design preferences for digital mental health interventions. Thematic analysis identified 5 key themes that offer novel and context-specific insights into how digital tools can be designed to address the unique challenges faced by paramedics. These findings contribute to a growing body of research advocating for trauma-informed, user-centered approaches in digital mental health interventions for emergency service workers.

First, participants emphasized that digital interventions need to be easy to use, with a simple, intuitive design that does not add to their cognitive load after stressful shifts. They highlighted the importance of quick access and ease of navigation, aligning with previous research indicating that digital mental health interventions must be designed for seamless usability to encourage engagement [34].

Second, participants expressed a strong preference for customization and personalization, with a tool that adapts to their individual coping styles, schedules, and emotional states. They noted that existing mental health resources often fail to address the distinct challenges of their role, reinforcing findings that user-centered digital interventions lead to higher adherence and satisfaction [60]. Participants also emphasized that their work schedules are unpredictable, meaning the intervention should be flexible, allowing them to engage with support at a time that suits them rather than being restricted to set hours or appointments.

Third, participants highlighted the importance of social connection and peer support, valuing the opportunity to connect with colleagues who understand the unique pressures of their role. They suggested that digital interventions should facilitate safe and structured peer interactions, aligning with research demonstrating the critical role of peer support in promoting resilience among first responders [61-63]. The ability to share experiences with others who have been through similar situations was seen as particularly beneficial, as it reduced the sense of isolation that many paramedics reported experiencing.

Fourth, concerns around confidentiality, privacy, and safety were significant, with participants voicing worries about data security and the need for absolute privacy to overcome fears of stigma or professional consequences. Many preferred an anonymous platform to reduce concerns about disclosure, echoing previous studies that highlight the importance of privacy in emergency workers’ engagement with mental health interventions [11]. They expressed particular concerns about their professional registration and career security, with some fearing that seeking support through formal occupational health routes could have negative consequences. A digital intervention designed with strong privacy protections was seen as a way to reduce these concerns and encourage engagement. However, with the need for anonymity comes an ethical requirement to ensure adequate support and safeguarding. Recent research on safeguarding of users of mental health apps has emphasized the importance of ensuring that platforms clearly highlight their limitations and capacities concerning identifying risk and referring individuals to outside care opportunities, in addition to any built-in interventions [64].

Finally, participants stressed the need for digital interventions to complement, rather than replace, human-led mental health support. While digital tools offer accessibility and discretion, participants recognized the value of professional guidance and structured follow-ups to ensure sustained engagement. Research supports a blended approach that integrates digital mental health tools with professional and peer-based support systems to maximize effectiveness [65]. Participants noted that digital interventions could serve as an important first step in seeking help, but that access to human support, whether through structured debriefs, therapy, or informal check-ins, remained a crucial component of effective mental health care. This aligns with earlier work whereby emergency workers have emphasized the importance of personal engagement in their training and education [66].

### Strengths, Limitations, and Future Directions

This study makes a valuable contribution to the existing body of knowledge by providing in-depth insights into paramedics’ experiences of workplace trauma and their perspectives on digital mental health interventions. A key strength of this study lies in the use of robust qualitative methodology, which enabled the generation of rich, contextually grounded findings. However, this study has some limitations. The sample consisted of 22 paramedics, which, while appropriate for qualitative research, may not fully capture the diverse experiences across different ambulance services and geographic locations. Additionally, participants self-selected into this study, which may have resulted in the inclusion of individuals who are more willing to engage in discussions about mental health, potentially limiting the generalizability of findings. Future research should aim to recruit a larger and more demographically diverse sample to explore a broader range of perspectives and experiences. Particular attention should be given to increasing representation of ethnically and culturally diverse paramedics, as this is essential not only for producing inclusive and representative findings but also for informing the effective transcreation and localization of digital interventions. This will contribute to more accessible, relevant, and equitable interventions, ultimately reducing disparities in mental health outcomes and supporting health equity across emergency service settings.

While purposive and snowball sampling allowed the research team to access a diverse range of paramedics with direct experience of workplace trauma, it is acknowledged that there is potential for self-selection bias. Participants who chose to take part may have been more open to discussing mental health or more comfortable with technology, compared to those who did not volunteer. As such, the sample may not fully reflect the perspectives of paramedics who are more reluctant to engage in conversations about mental health or who may be more skeptical about digital interventions. This should be considered when interpreting the findings, particularly concerning the anticipated uptake of digital tools across the broader paramedic workforce.

Another limitation is that this study focused solely on paramedics, whereas other emergency responders, such as firefighters and police officers, also experience similar mental health challenges. Comparative studies across different frontline professions would help identify both shared and profession-specific needs for digital interventions. Additionally, this study explored user preferences but did not evaluate an actual digital intervention. The research team is currently undertaking a program of work focused on the co-design, development, and real-world testing of a digital tool, Sentinel, to evaluate its feasibility, usability, and impact on mental health outcomes among first responders [67]. By incorporating paramedic feedback throughout the development process, Sentinel aims to address usability, personalization, peer connectivity, and confidentiality concerns. The intervention will be piloted with paramedics to evaluate its effectiveness and integration into existing mental health support systems. Furthermore, customized versions of Sentinel will be expanded to support other frontline professionals, including firefighters, police officers, and emergency health care staff, recognizing both the common and unique stressors they encounter. Additionally, the platform will offer resources for the families of frontline workers, acknowledging their vital role in providing support and the importance of addressing their specific needs to help foster stronger community and familial resilience [68]. Future studies will examine how these adaptations enhance mental health support across various high-pressure occupational settings, leading to a scalable, evidence-based intervention for frontline workers [69].

The findings of the current study have clear implications for ambulance services and policymakers seeking to improve the mental health of frontline staff. Services should consider investing in digital tools that are co-designed with paramedics, offer confidentiality, and are accessible outside of traditional working hours. Importantly, such tools should not operate in isolation but be embedded within wider organizational strategies that promote psychological safety and reduce stigma around help-seeking. Embedding trauma-informed support into routine practice, through education, peer-support systems, and leadership engagement, can help foster a culture where mental health conversations are normalized and proactively supported. Policy makers can play a key role in endorsing the development and adoption of such interventions across emergency services, ensuring that funding mechanisms and regulatory guidance align with the psychological needs of high-risk occupational groups.

While future technological innovations hold promise, the immediate priority lies in developing usable, human-centered digital support grounded in the lived experience of paramedics. Artificial intelligence, wearable data, and real-time analytics may offer longer-term opportunities to enhance personalization, tailor content delivery, and monitor distress signals [70]. However, any integration of such technologies must be approached cautiously, with strong attention to ethical considerations, including data privacy, informed consent, and algorithmic bias [71]. Crucially, future research must ensure that artificial intelligence–driven tools remain grounded in cocreation and usability principles, as interventions designed with direct user input are more likely to be trusted and effective in real-world settings [72,73].

To support scalability and implementation, structured frameworks such as RE-AIM (Reach, Effectiveness, Adoption, Implementation, and Maintenance), the UK Medical Research Council guidance on complex interventions, or the technology acceptance model could be used to guide development and evaluation [74-78]. These frameworks can help developers attend to issues of accessibility, human interaction, and long-term sustainability, ensuring that digital interventions are both usable and adaptable to diverse frontline professions. Ultimately, while innovation in digital health is advancing rapidly, this study underscores the critical value of listening to paramedics themselves. Future work should continue to center on the voices of frontline workers to ensure that digital mental health tools are responsive, acceptable, and fit for purpose in the demanding realities of emergency service work.

### Conclusions

This study highlights the importance of designing digital mental health interventions that are user-friendly, personalized, confidential, and integrated with existing support services. The findings provide critical insights into the specific needs of paramedics, particularly concerning their frequent exposure to workplace trauma, and outline key design considerations for digital mental health tools. Given the high levels of trauma-related psychological distress among paramedics, tailored digital solutions have the potential to offer accessible, flexible, and trauma-informed support. Future research should prioritize cocreating and rigorously evaluating digital interventions with paramedics and other emergency responders to ensure real-world usability and clinical relevance. By integrating end user feedback and addressing the psychological impact of trauma directly, digital mental health tools can enhance engagement, promote well-being, and provide frontline workers with timely support to navigate the emotional demands of their work. Through ongoing collaboration and refinement, trauma-informed digital interventions have the potential to transform the accessibility and effectiveness of mental health care for paramedics and other frontline professionals.

## Abbreviations

RE-AIM: Reach, Effectiveness, Adoption, Implementation, and Maintenance
